# Supplementary material for: Computational modeling demonstrates that glioblastoma cells can survive spatial environmental challenges through exploratory adaptation
Source: Nat Commun. 2019 Dec 13;10:5704. doi: 10.1038/s41467-019-13726-w (PMC6911112; doi:10.1038/s41467-019-13726-w)
Supplement: Supplementary file 2 — Supplementary Information [file 41467_2019_13726_MOESM2_ESM.pdf]

Computational modeling demonstrates that glioblastoma cells can survive spatial environmental challenges through exploratory adaptation. Celiku el at.

Supplementary Figure 1

A)

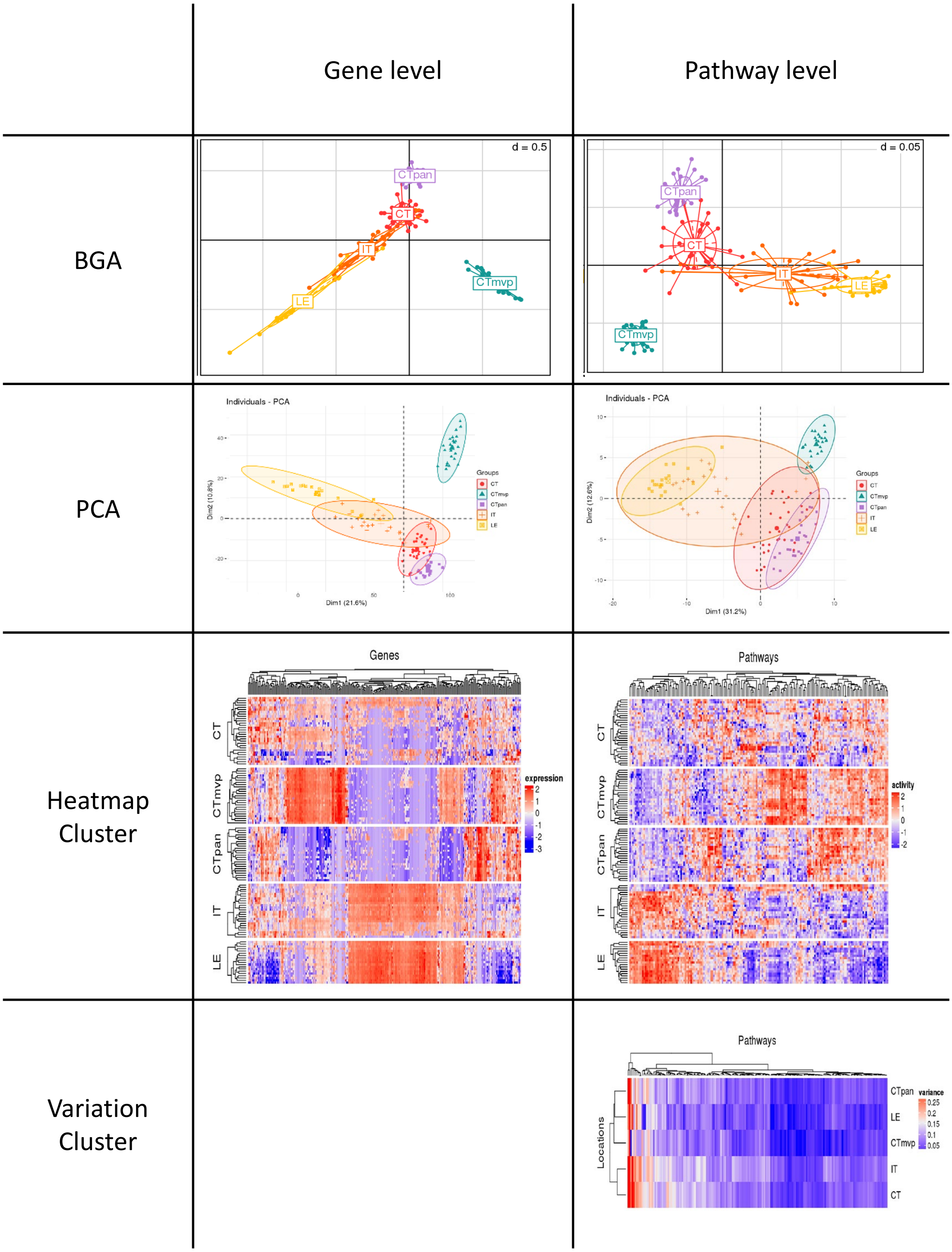

B)

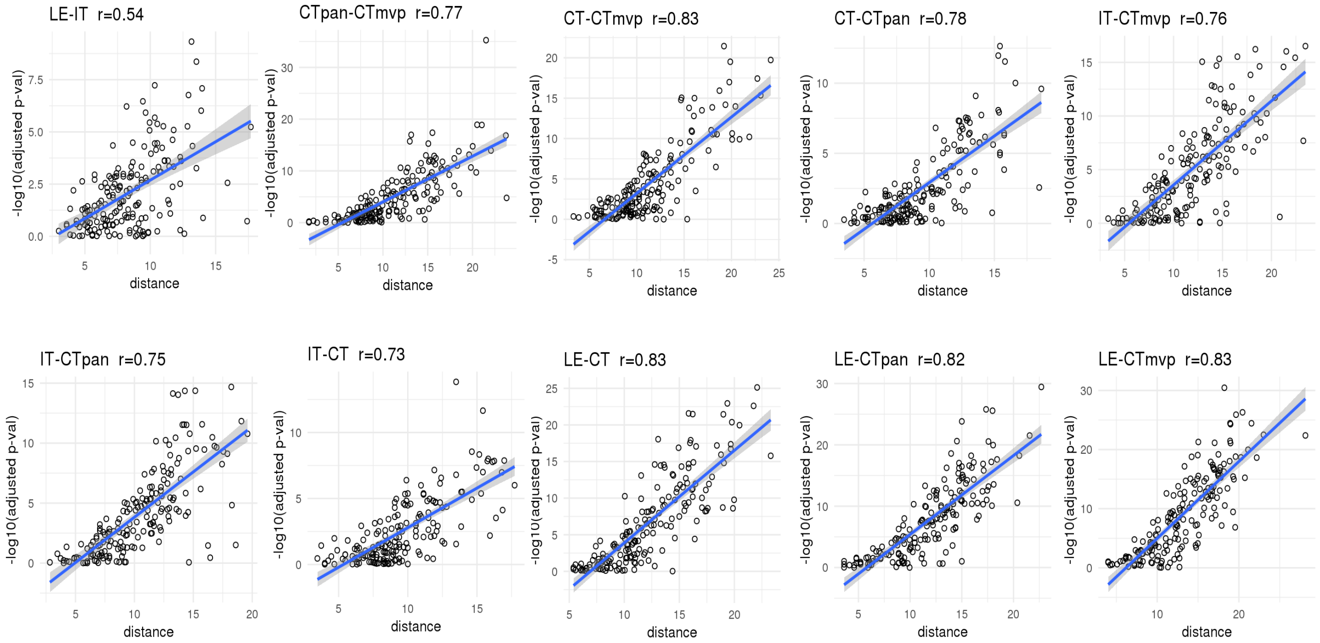
LE-IT r=0.54
CTpan-CTmvp r=0.77
CT-CTmvp r=0.83
CT-CTpan r=0.78
IT-CTmvp r=0.76
IT-CTpan r=0.75
IT-CT r=0.73
LE-CT r=0.83
LE-CTpan r=0.82
LE-CTmvp r=0.83
-log10(adjusted p-val)
distance

C)

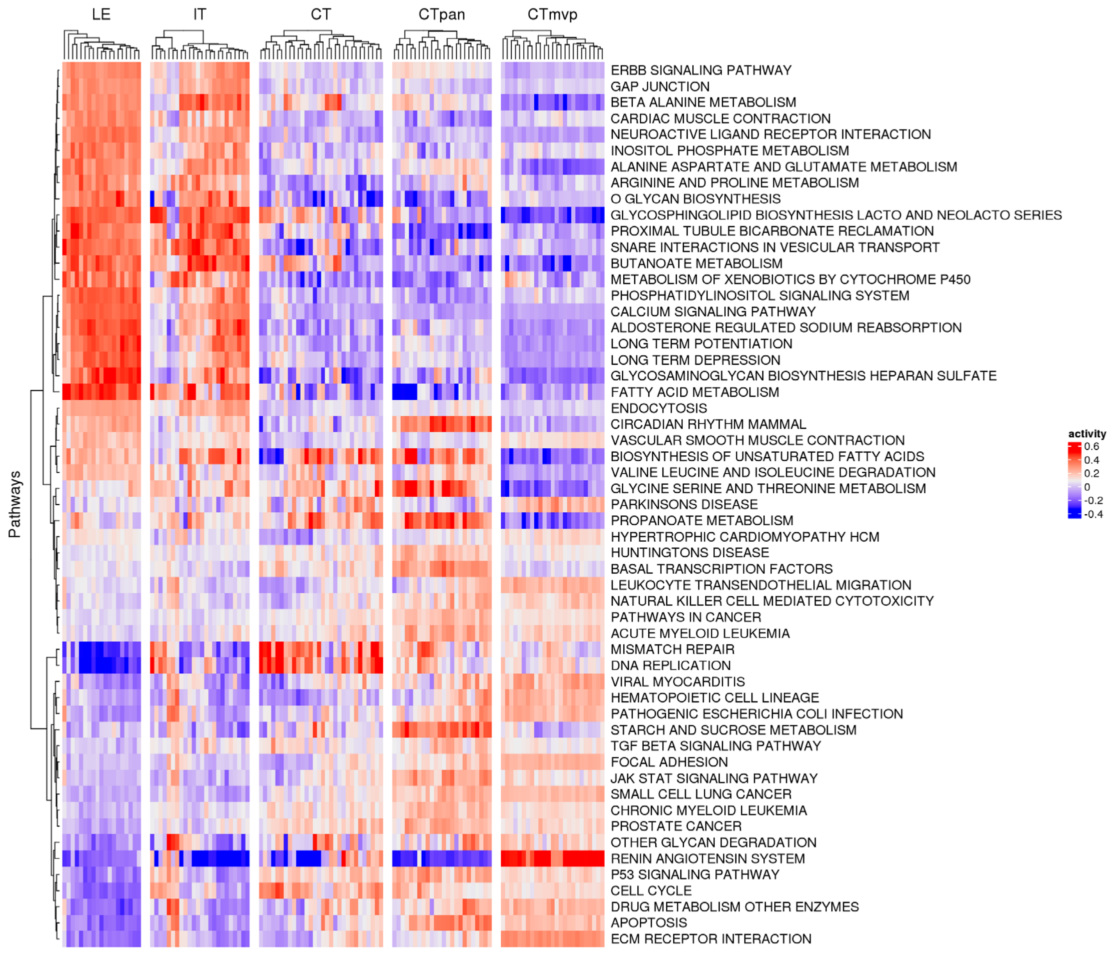
LE
IT
CT
CTpan
CTmvp
Pathways
ERBB SIGNALING PATHWAY
GAP JUNCTION
BETA ALANINE METABOLISM
CARDIAC MUSCLE CONTRACTION
NEUROACTIVE LIGAND RECEPTOR INTERACTION
INOSITOL PHOSPHATE METABOLISM
ALANINE ASPARTATE AND GLUTAMATE METABOLISM
ARGININE AND PROLINE METABOLISM
O GLYCAN BIOSYNTHESIS
GLYCOSPHINGOLIPID BIOSYNTHESIS LACTO AND NEOLACTO SERIES
PROXIMAL TUBULE BICARBONATE RECLAMATION
SNARE INTERACTIONS IN VESICULAR TRANSPORT
BUTANOATE METABOLISM
METABOLISM OF XENOBIOTICS BY CYTOCHROME P450
PHOSPHATIDYLINOSITOL SIGNALING SYSTEM
CALCIUM SIGNALING PATHWAY
ALDOSTERONE REGULATED SODIUM REABSORPTION
LONG TERM POTENTIATION
LONG TERM DEPRESSION
GLYCOSAMINOGLYCAN BIOSYNTHESIS HEPARAN SULFATE
FATTY ACID METABOLISM
ENDOCYTOSIS
CIRCADIAN RHYTHM MAMMAL
VASCULAR SMOOTH MUSCLE CONTRACTION
BIOSYNTHESIS OF UNSATURATED FATTY ACIDS
VALINE LEUCINE AND ISOLEUCINE DEGRADATION
GLYCINE SERINE AND THREONINE METABOLISM
PARKINSONS DISEASE
PROPANOATE METABOLISM
HYPERTROPHIC CARDIOMYOPATHY HCM
HUNTINGTONS DISEASE
BASAL TRANSCRIPTION FACTORS
LEUKOCYTE TRANSENDOTHELIAL MIGRATION
NATURAL KILLER CELL MEDIATED CYTOTOXICITY
PATHWAYS IN CANCER
ACUTE MYELOID LEUKEMIA
MISMATCH REPAIR
DNA REPLICATION
VIRAL MYOCARDITIS
HEMATOPOIETIC CELL LINEAGE
PATHOGENIC ESCHERICHIA COLI INFECTION
STARCH AND SUCROSE METABOLISM
TGF BETA SIGNALING PATHWAY
FOCAL ADHESION
JAK STAT SIGNALING PATHWAY
SMALL CELL LUNG CANCER
CHRONIC MYELOID LEUKEMIA
PROSTATE CANCER
OTHER GLYCAN DEGRADATION
RENIN ANGIOTENSIN SYSTEM
P53 SIGNALING PATHWAY
CELL CYCLE
DRUG METABOLISM OTHER ENZYMES
APOPTOSIS
ECM RECEPTOR INTERACTION
activity
0.6
0.4
0.2
0
-0.2
-0.4

Supplementary Figure 1: Initial data analysis

A) Differences between gene and pathway levels characterizing location-based phenotypes. The spatial organization is apparent in a Between Group Analysis (BGA), Principal Component Analysis (PCA), and hierarchical clustering in expression heatmap plots. All pathway-level plots display overlap between locations, suggesting a continuous transition along the spatial trajectories; the overlap is less visible in the gene-level plots. From BGA and the heatmap clusters, it is apparent that the CT phenotype is the center of all locations. From CT, there are 3 closest locations, CTmvp, CTpan, and IT, which are distinct from each other. LE is the next closest location starting from IT. Thus, we have three immediate trajectories, CT->IT->LE, CT->CTmvp, CT->CTpan. Moreover, distinct patterns of variation in location phenotypes are also apparent: CT and IT show higher variance in the activity distributions of a number of pathways, and similar patterns of variation compared to the other locations.

B) Correlation between PDD and t-test. We estimated the significance of pathway activity variations between two locations using unpaired, two-sided t-test (BH-adjusted p-values) and found good correlation with the PDD values (Pearson= 0.5-0.7, Table S1).

C) Heatmap of 55 most significant pathways. Clustering groups of samples based on their pathway activity patterns can show the differences in location-based phenotype. Here we listed the 55 most significant pathways for biological interpretation.

Supplementary Figure 2

A)

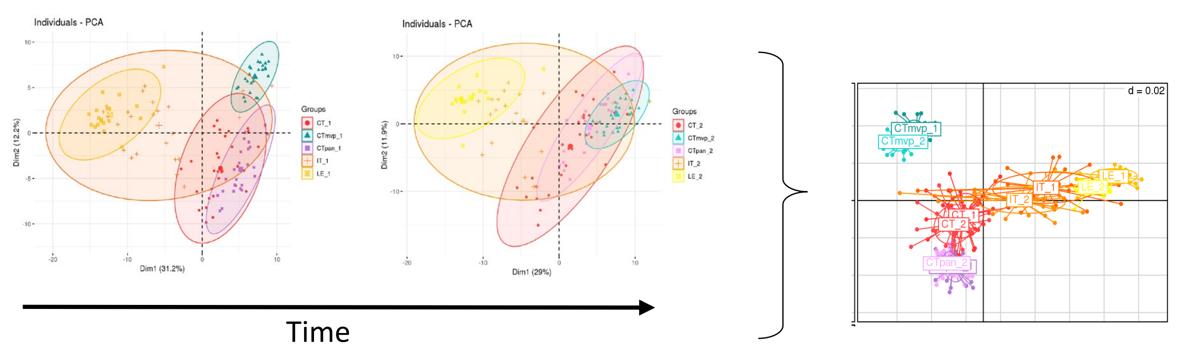

B)

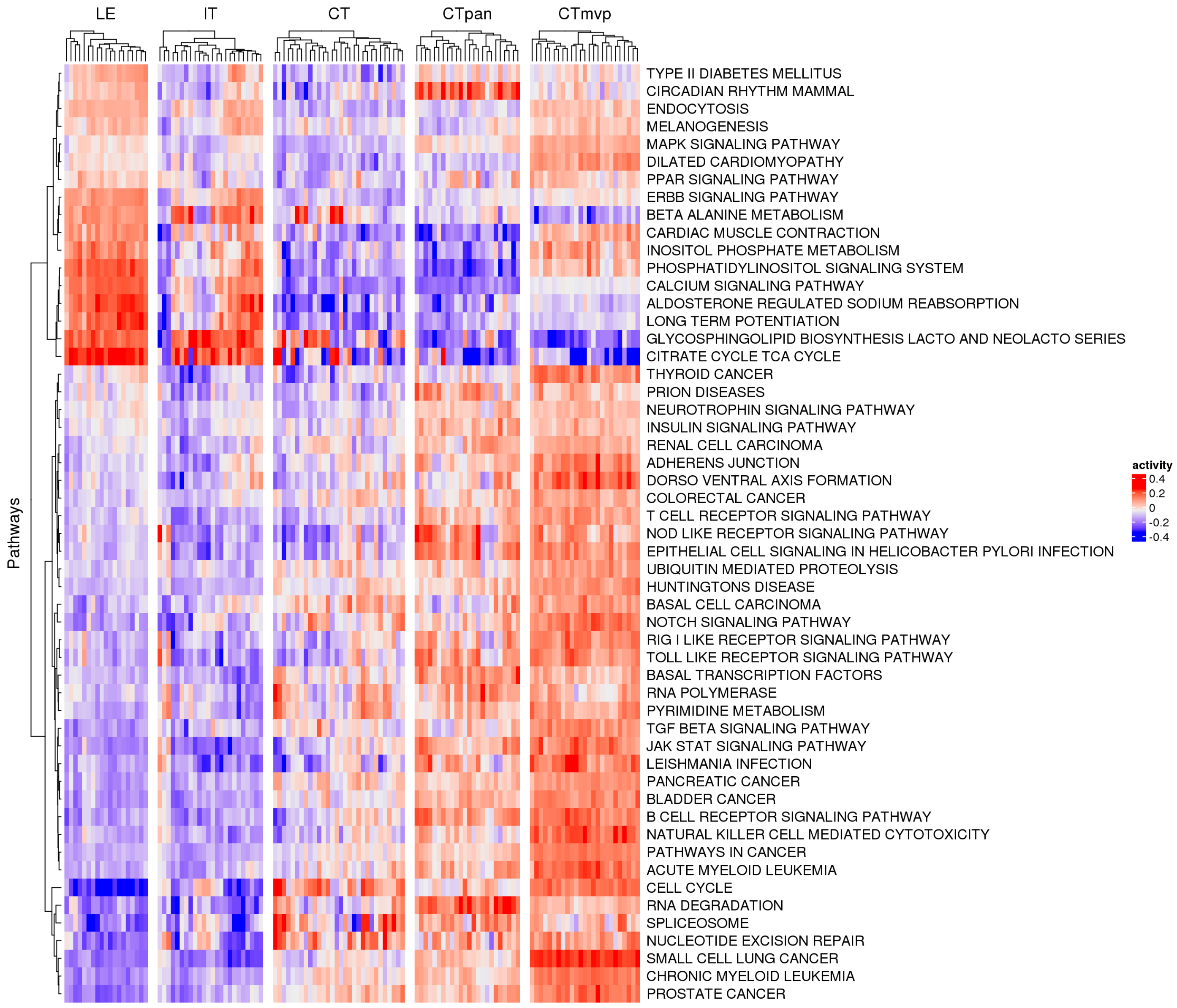

Supplementary Figure 2: Simulation results of intrinsic model (t=[0,1000])

A) Phenotype differences based on intrinsic model (with no exploratory adaptation, $dJ = 0$). PCA visualization of the temporal changes in location-based phenotype between the original data (time =0), and after 1,000 time steps of simulation.

B) Heatmap of pathways whose activity changes significantly between the initial and final time points

Supplementary Figure 3

Subplots A-C provide an example of a single sample dynamics, CT->CTmvp
Subplots D-F provide an example of a single sample dynamics, CT->LE

A)

Intrinsic behavior ($dJ = 0$) EA behavior ($dJ > 0$)

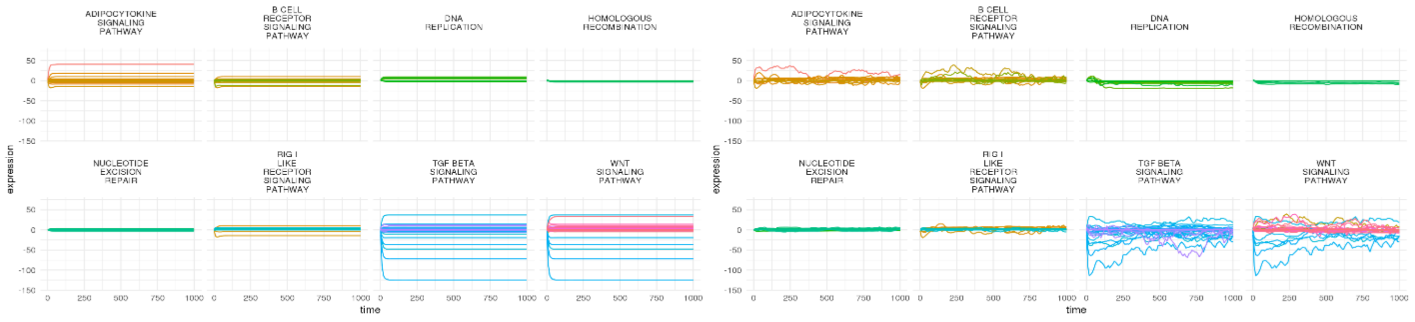

B)

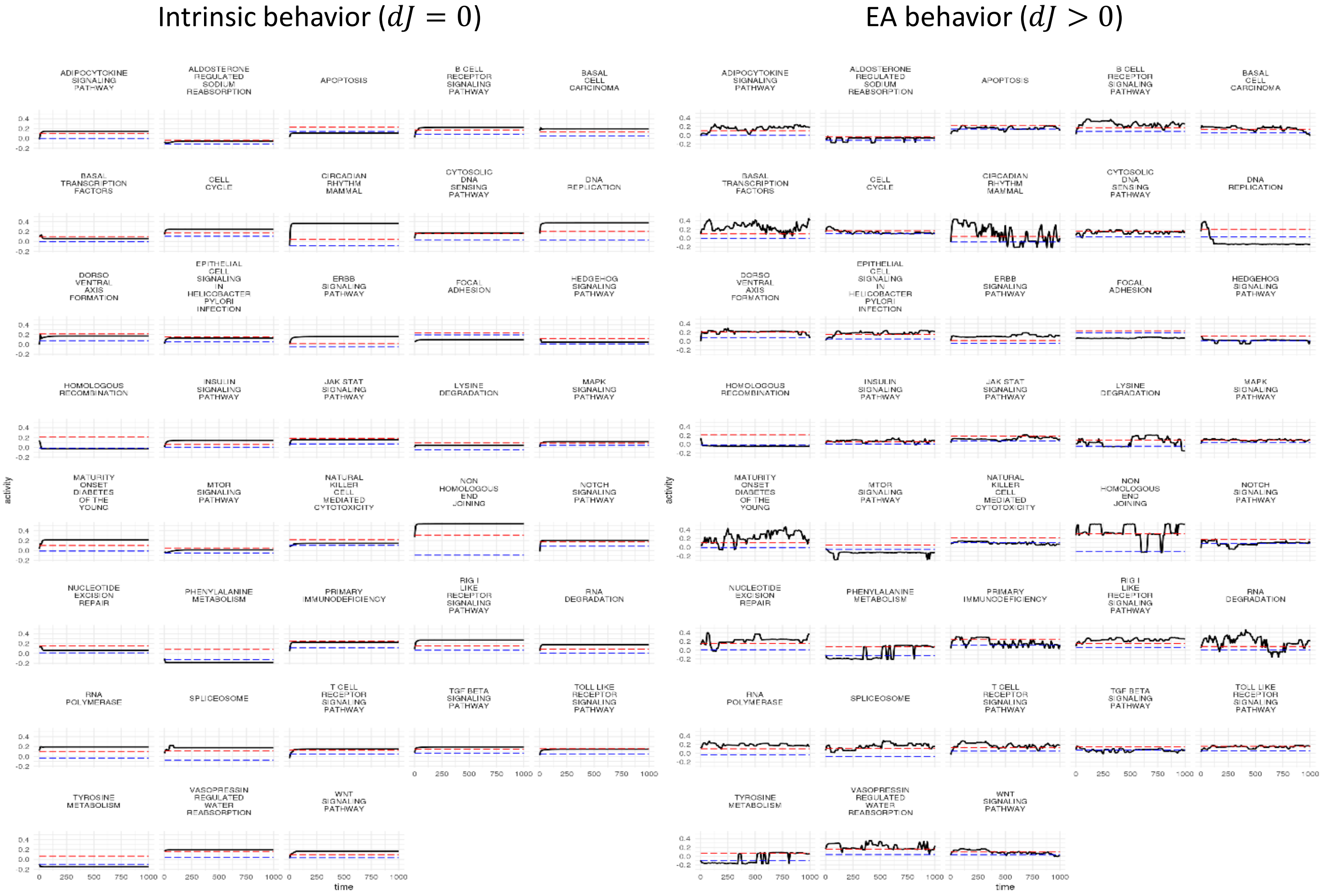
Intrinsic behavior ($dJ = 0$)
EA behavior ($dJ > 0$)
ADIPOCYTOKINE SIGNALING PATHWAY
ALDOSTERONE REGULATED SODIUM REABSORPTION
APOPTOSIS
B CELL RECEPTOR SIGNALING PATHWAY
BASAL CELL CARCINOMA
BASAL TRANSCRIPTION FACTORS
CELL CYCLE
CIRCADIAN RHYTHM MAMMAL
CYTOSOLIC DNA SENSING PATHWAY
DNA REPLICATION
DORSO VENTRAL AXIS FORMATION
EPITHELIAL CELL SIGNALING IN HELICOBACTER PYLORI INFECTION
ERBB SIGNALING PATHWAY
FOCAL ADHESION
HEDGEHOG SIGNALING PATHWAY
HOMOLOGOUS RECOMBINATION
INSULIN SIGNALING PATHWAY
JAK STAT SIGNALING PATHWAY
LYSINE DEGRADATION
MAPK SIGNALING PATHWAY
MATURITY ONSET DIABETES OF THE YOUNG
MTOR SIGNALING PATHWAY
NATURAL KILLER CELL MEDIATED CYTOTOXICITY
NON HOMOLOGOUS END JOINING
NOTCH SIGNALING PATHWAY
NUCLEOTIDE EXCISION REPAIR
PHENYLALANINE METABOLISM
PRIMARY IMMUNODEFICIENCY
RIG I LIKE RECEPTOR SIGNALING PATHWAY
RNA DEGRADATION
RNA POLYMERASE
SPLICEOSOME
T CELL RECEPTOR SIGNALING PATHWAY
TGF BETA SIGNALING PATHWAY
TOLL LIKE RECEPTOR SIGNALING PATHWAY
TYROSINE METABOLISM
VASOPRESSIN REGULATED WATER REABSORPTION
WNT SIGNALING PATHWAY
activity
time
0 250 500 750 1000
0.4 0.2 0.0 -0.2

C)

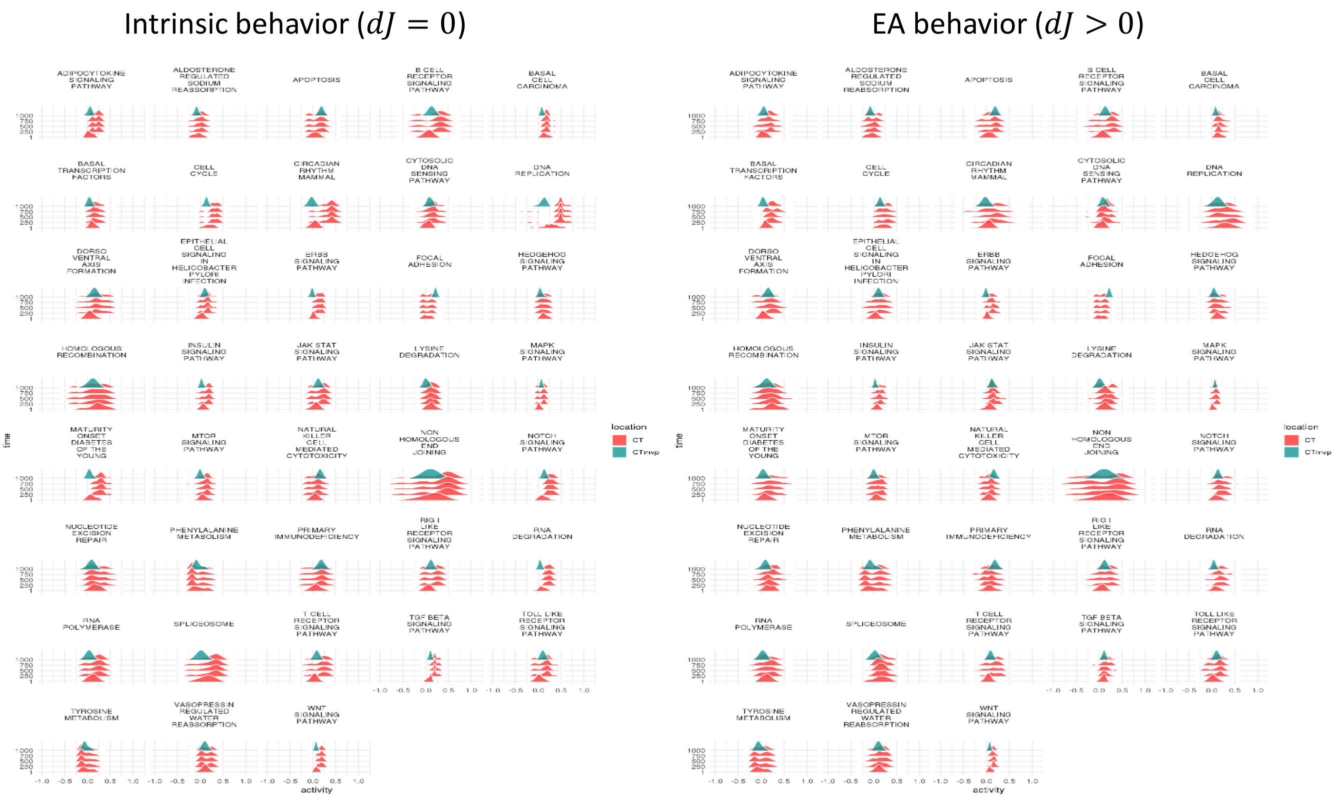
Intrinsic behavior ($dJ = 0$)
EA behavior ($dJ > 0$)
ADIPOCYTOKINE SIGNALING PATHWAY
ALDOSTERONE REGULATED SODIUM REABSORPTION
APOPTOSIS
B CELL RECEPTOR SIGNALING PATHWAY
BASAL CELL CARCINOMA
BASAL TRANSCRIPTION FACTORS
CELL CYCLE
CIRCADIAN RHYTHM MAMMAL
CYTOSOLIC DNA SENSING PATHWAY
DNA REPLICATION
DORSO VENTRAL AXIS FORMATION
EPITHELIAL CELL SIGNALING IN HELICOBACTER PYLORI INFECTION
ERBB SIGNALING PATHWAY
FOCAL ADHESION
HEDGEHOG SIGNALING PATHWAY
HOMOLOGOUS RECOMBINATION
INSULIN SIGNALING PATHWAY
JAK STAT SIGNALING PATHWAY
LYSINE DEGRADATION
MAPK SIGNALING PATHWAY
MATURITY ONSET DIABETES OF THE YOUNG
MTOR SIGNALING PATHWAY
NATURAL KILLER CELL MEDIATED CYTOTOXICITY
NON HOMOLOGOUS END JOINING
NOTCH SIGNALING PATHWAY
NUCLEOTIDE EXCISION REPAIR
PHENYLALANINE METABOLISM
PRIMARY IMMUNODEFICIENCY
RIG I LIKE RECEPTOR SIGNALING PATHWAY
RNA DEGRADATION
RNA POLYMERASE
SPLICEOSOME
T CELL RECEPTOR SIGNALING PATHWAY
TGF BETA SIGNALING PATHWAY
TOLL LIKE RECEPTOR SIGNALING PATHWAY
TYROSINE METABOLISM
VASOPRESSIN REGULATED WATER REABSORPTION
WNT SIGNALING PATHWAY
time
activity
location
CT
CTmvp

D)

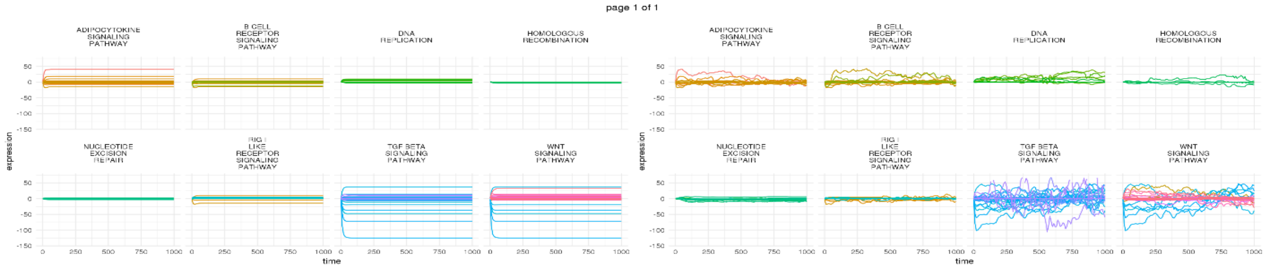
page 1 of 1
ADIPOCYTOKINE SIGNALING PATHWAY
B CELL RECEPTOR SIGNALING PATHWAY
DNA REPLICATION
HOMOLOGOUS RECOMBINATION
NUCLEOTIDE EXCISION REPAIR
RIG I LIKE RECEPTOR SIGNALING PATHWAY
TGF BETA SIGNALING PATHWAY
WNT SIGNALING PATHWAY
expression
time

E)

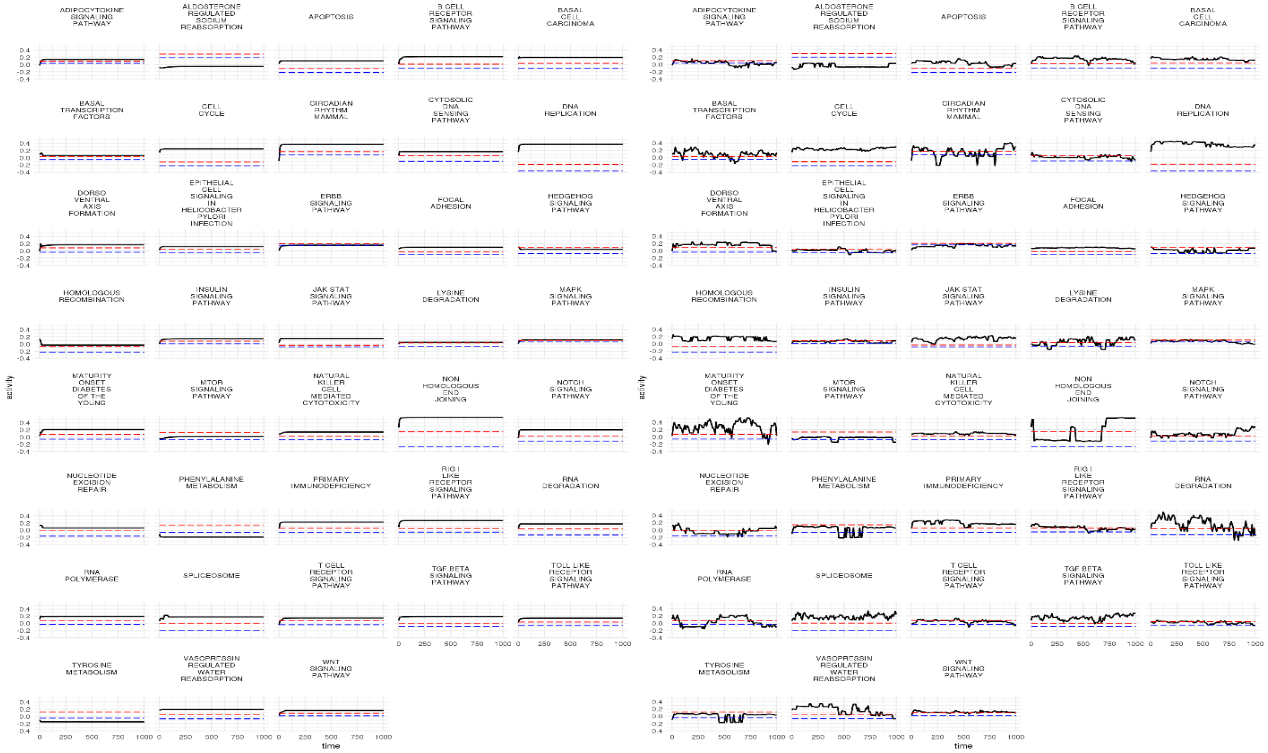
ADIPOCYTOKINE SIGNALING PATHWAY
ALDOSTERONE REGULATED SODIUM REABSORPTION
APOPTOSIS
B CELL RECEPTOR SIGNALING PATHWAY
BASAL CELL CARCINOMA
BASAL TRANSCRIPTION FACTORS
CELL CYCLE
CIRCADIAN RHYTHM MAMMAL
CYTOSOLIC DNA SENSING PATHWAY
DNA REPLICATION
DORSO VENTRAL AXIS FORMATION
EPITHELIAL CELL SIGNALING IN HELICOBACTER PYLORI INFECTION
ERBB SIGNALING PATHWAY
FOCAL ADHESION
HEDGEHOG SIGNALING PATHWAY
HOMOLOGOUS RECOMBINATION
INSULIN SIGNALING PATHWAY
JAK STAT SIGNALING PATHWAY
LYSINE DEGRADATION
MAPK SIGNALING PATHWAY
MATURITY ONSET DIABETES OF THE YOUNG
MTOR SIGNALING PATHWAY
NATURAL KILLER CELL MEDIATED CYTOTOXICITY
NON HOMOLOGOUS END JOINING
NOTCH SIGNALING PATHWAY
NUCLEOTIDE EXCISION REPAIR
PHENYLALANINE METABOLISM
PRIMARY IMMUNODEFICIENCY
RIG I LIKE RECEPTOR SIGNALING PATHWAY
RNA DEGRADATION
RNA POLYMERASE
SPLICEOSOME
T CELL RECEPTOR SIGNALING PATHWAY
TGF BETA SIGNALING PATHWAY
TOLL LIKE RECEPTOR SIGNALING PATHWAY
TYROSINE METABOLISM
VASOPRESSIN REGULATED WATER REABSORPTION
WNT SIGNALING PATHWAY
activity
time

F)

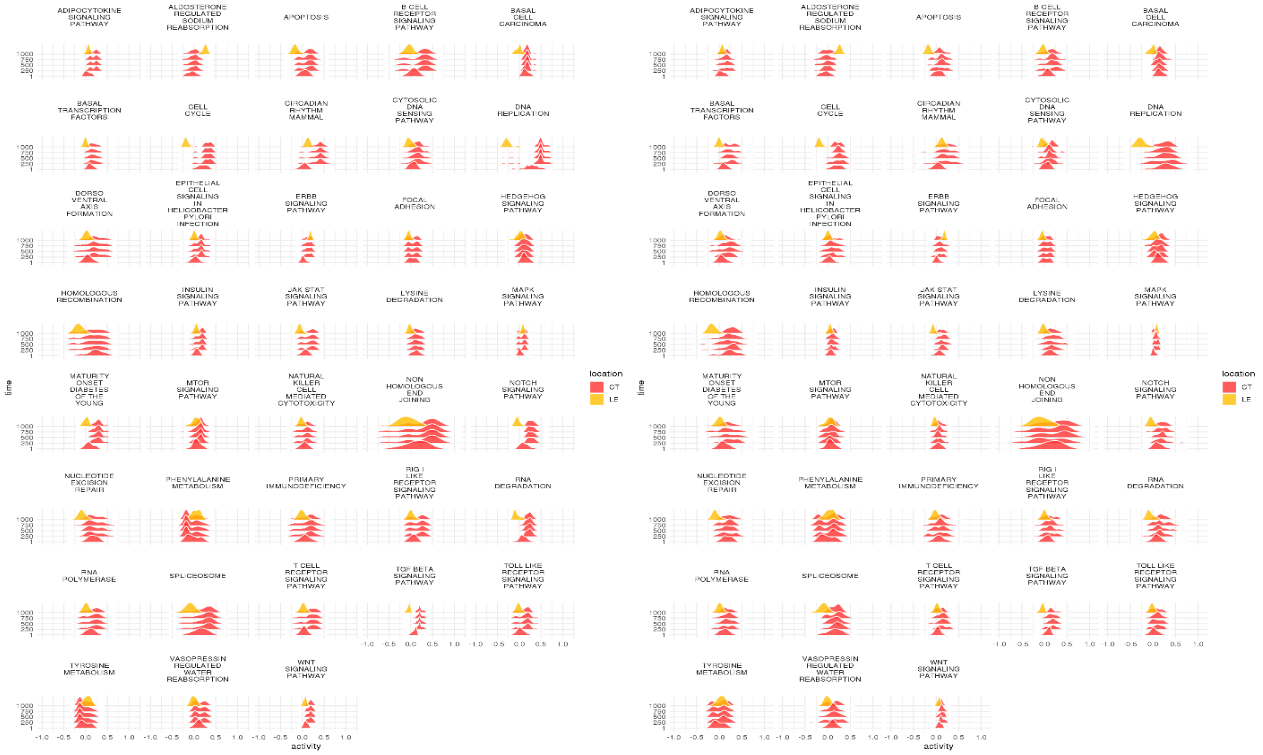
location
CT
LE
time
activity

Supplementary Figure 3: Two examples of single sample dynamics

Subplots A-C provide an example of the dynamics of a single sample moving from CT to CTmvp
Subplots D-F provide an example of the dynamics of a single sample moving from CT to LE

A) Comparisons between the intrinsic and EA simulations of eight example pathways are shown. For each pathway, several simulation results of different tumor samples were plotted to illustrate the stochasticity in this process.

We investigated whether our model could explain the dynamical process of transitioning between two phenotypes along the spatial trajectories. For a given location pair, for example CT→CTmvp, we simulated for each sample *s* from CT, the phenotypes that can be obtained through exploratory adaption (EA) over a given time interval [*initial, final*]. We computed the distance of *s*'s phenotypes at time point *final* from CTmvp (as the sum of the distances of the pathway activities of *s* from the 10-90% percentile of the target CTmpv pathway distributions as detailed in Methods). The degree to which the samples of a location reduced their distance to their target phenotype measures the degree to which exploratory adaption is responsible for the differences in phenotypes. We compared these distances with those obtained from simulation of intrinsic behavior ($dJ = 0$), and when these distances were not smaller than those obtained in the intrinsic case, we concluded that exploratory adaption is not necessary to explain those phenotype differences.

B) Dynamics of pathway activities. All pathway activities of a single tumor sample (at time=0) are shown for two cases of intrinsic and EA simulations.

C) All pathway activities of all tumor samples (as time=0) are shown are shown for the two cases of intrinsic and EA simulations.

D-F) Similar plots as in (A-C) that illustrate the dynamics of a single sample moving from CT->CTmvp
