# Supplementary material for: Computational modeling demonstrates that glioblastoma cells can survive spatial environmental challenges through exploratory adaptation
Source: Nat Commun. 2019 Dec 13;10:5704. doi: 10.1038/s41467-019-13726-w (PMC6911112; doi:10.1038/s41467-019-13726-w)
Supplement: Supplementary file 3 — Description of Additional Supplementary Files [file 41467_2019_13726_MOESM3_ESM.pdf]

Description of Additional Supplementary Files

Supplementary Data 1: Intrinsic adaptation

sheet1	Gene set
sheet2	Ivy-Gap tumor samples' information
sheet3	Pathway Distribution Distance (PDD) and t-test
sheet4	Pathways passing cutoff for each comparison Pval<1e-06
sheet5	Pearson Correlation of PDD with -log10(p-adjust) of t-test
sheet6	Initial global distribution distance (GDD) between pair of locations
sheet7	Permutation test of GDD

cellular tumor (CT), leading edge (LE), infiltrating tumor (IT), pseudopalisading region around necrosis (CTpan), and microvascular proliferation (CTmvp). PDD= Pathway Distribution Distance. GD=Global Distribution Distance. NP=number of pathways that have statistically significant differences

Supplementary Data 2: Exploratory adaptation

sheet1	Intrinsic Pathway distance between t=[0,1000] & t-test
sheet2	Intrinsic Pathways passing cutoff for each comparison Pval<1e-03:
sheet3	Intrinsic Correlation between PD and t-test
sheet4	Intrinsic global distribution distance between t=1000 and t=0
sheet5	Exploratory model - GD results
sheet6	Permutation test of GD
sheet7	Intermediate phenotypes, dj>0, D=0.1
sheet8	Discretizing the continuous phenotype trajectories (a therapeutic roadmap)

Intrinsic model t=[0,1000] - no exploratory adaptation ability

Supplementary Movies 1-2: Intrinsic vs. exploratory adaptation from CT to CTmvp

Focusing on the case of adaptation from CT to CTmvp, we examined in detail two GBM relevant pathways, DNA replication and B cell receptor signaling pathways; the animation in Supplementary Movie 1 illustrates intrinsic adaptation and Supplementary Movie 2 illustrates exploratory adaptation.
